# Identification of cuproptosis-related biomarkers and analysis of immune infiltration in allograft lung ischemia-reperfusion injury

**DOI:** 10.3389/fmolb.2023.1269478

**Published:** 2023-11-21

**Authors:** Jianying Qin, Xiaoyue Xiao, Silin Li, Ning Wen, Ke Qin, Haibin Li, Jihua Wu, Bing Lu, Minghu Li, Xuyong Sun

**Affiliations:** ^1^ Transplant Medical Center, The Second Affiliated Hospital of Guangxi Medical University, Nanning, China; ^2^ Guangxi Clinical Research Center for Organ Transplantation, Nanning, China; ^3^ Guangxi Key Laboratory of Organ Donation and Transplantation, Nanning, China; ^4^ Department of Pathology, The Fifth Affiliated Hospital of Guangxi Medical University, The First People’s Hospital of Nanning, Nanning, China

**Keywords:** cuproptosis, ischemia-reperfusion injury, allograft lung, biomarkers, immune infiltration

## Abstract

**Background:** Allograft lung ischemia-reperfusion injury (ALIRI) is a major cause of early primary graft dysfunction and poor long-term survival after lung transplantation (LTx); however, its pathogenesis has not been fully elucidated. Cell death is a mechanism underlying ALIRI. Cuproptosis is a recently discovered form of programmed cell death. To date, no studies have been conducted on the mechanisms by which cuproptosis-related genes (CRGs) regulate ALIRI. Therefore, we explored the potential biomarkers related to cuproptosis to provide new insights into the treatment of ALIRI.

**Materials and methods:** Datasets containing pre- and post-LTx lung biopsy samples and CRGs were obtained from the GEO database and previous studies. We identified differentially expressed CRGs (DE-CRGs) and performed functional analyses. Biomarker genes were selected using three machine learning algorithms. The ROC curve and logistic regression model (LRM) of these biomarkers were constructed. CIBERSORT was used to calculate the number of infiltrating immune cells pre- and post-LTx, and the correlation between these biomarkers and immune cells was analyzed. A competing endogenous RNA network was constructed using these biomarkers. Finally, the biomarkers were verified in a validation set and a rat LTx model using qRT-PCR and Western blotting.

**Results:** Fifteen DE-CRGs were identified. GO analysis revealed that DE-CRGs were significantly enriched in the mitochondrial acetyl-CoA biosynthetic process from pyruvate, protein lipoylation, the tricarboxylic acid (TCA) cycle, and copper-transporting ATPase activity. KEGG enrichment analysis showed that the DE-CRGs were mainly enriched in metabolic pathways, carbon metabolism, and the TCA cycle. *NFE2L2*, *NLRP3*, *LIPT1*, and *MTF1* were identified as potential biomarker genes. The AUC of the ROC curve for each biomarker was greater than 0.8, and the LRM provided an excellent classifier with an AUC of 0.96. These biomarkers were validated in another dataset and a rat LTx model, which exhibited good performance. In the CIBERSORT analysis, differentially expressed immune cells were identified, and the biomarkers were associated with the immune cells.

**Conclusion:** *NFE2L2*, *NLRP3*, *LIPT1*, and *MTF1* may serve as predictors of cuproptosis and play an important role in the pathogenesis of cuproptosis in ALIRI.

## 1 Introduction

Lung transplantation (LTx) is the only viable treatment for end-stage lung disease (Chen-Yoshikawa et al., 2020). However, due to early primary graft dysfunction (PGD) and late chronic lung allograft dysfunction (CLAD) post-LTx, the 1- and 5-year survival rates of recipients are only 85% and 59%, respectively (Chambers et al., 2017; Chambers et al., 2021). PGD is not only the leading cause of mortality in the perioperative period but also a risk factor for CLAD (Chacon-Alberty et al., 2023). PGD arises predominantly from allograft lung ischemia-reperfusion injury (ALIRI), which can promote chronic rejection and drive CLAD (Watanabe et al., 2019). Therefore, exploring the mechanism by which ALIRI reduces the morbidity of PGD and CLAD remains a key research focus. Allograft lungs are inevitably subject to ALIRI during organ harvesting, cold preservation, and transplantation. Recent studies have shown that cell death and inflammatory responses are the two major mechanisms underlying ALIRI (Wong and Liu, 2021; Capuzzimati et al., 2022).

Cuproptosis, a new form of programmed cell death (PCD), differs from previously reported forms of cell death, such as apoptosis, ferroptosis, and necroptosis. In this process, intracellular copper-ion overload can target and bind to lipoylated components of the tricarboxylic acid (TCA) cycle, resulting in the aggregation of lipoacylated proteins and the loss of iron-sulfur cluster proteins, leading to proteotoxic stress and, ultimately, cell death (Tsvetkov et al., 2022). Researchers have also identified cuproptosis-related genes (CRGs) (Tsvetkov et al., 2022). Previous research has shown that a Cu imbalance is associated with a wide range of pathological conditions, including neurodegenerative diseases, cancer, and cardiovascular disease (Chen et al., 2022). An earlier study found that increasing the cardiac copper content can enhance ischemic reperfusion injury (IRI) (Powell et al., 1991). However, a recent study also demonstrated that Cu may have protective effects against IRI in the heart and lungs of rats (Tural et al., 2020). These findings demonstrate the important role of copper in IRI; however, its effects and underlying mechanisms need to be explored further. As a newly identified form of PCD, the role of cuproptosis in the pathogenesis of ALIRI and the immune response against ALIRI remains unclear, and its potential as a therapeutic target for ALIRI is yet to be evaluated. Therefore, it is necessary to further explore CRGs that are associated with ALIRI, which may act as new targets for treating ALIRI.

To explore the role of cuproptosis in the pathogenesis of ALIRI and the immune response against ALIRI, we used the Gene Expression Omnibus (GEO) database to analyze the differentially expressed CRGs (DE-CRGs) between pre- and post-LTx human allograft lung samples. Cuproptosis-related biomarkers were screened from DE-CRGs using three machine learning (ML) algorithms, including the least absolute shrinkage and selection operator (LASSO), support vector machine recursive feature elimination (SVM-RFE), and random forest (RF) algorithms. We investigated the infiltrating immune cells in the datasets and their correlation with these biomarkers. A competing endogenous RNA (ceRNA) network was constructed using these biomarkers. Finally, the biomarkers were verified using another independent dataset, and quantitative real-time PCR (qRT-PCR) and Western blotting (WB) in allograft lung tissues from a rat LTx model. This study is the first to investigate the role of cuproptosis in ALIRI, which may provide more insights into the role of cuproptosis in ALIRI, along with the diagnosis and treatment of ALIRI. This study was conducted in accordance with the Declaration of Helsinki (revised in 2013). The workflow of this study is illustrated in Supplementary Figure S1.

## 2 Materials and methods

### 2.1 Acquisition of datasets and CRGs

Two microarray datasets (GSE145989 and GSE127003) were obtained from the GEO database (https://www.ncbi.nlm.nih.gov/geo/). These datasets were obtained from human lung allograft biopsy samples. Paired samples (collected at the end of cold ischemia and after 2 h of reperfusion, following transplantation) were included. Approximately 51 ischemic vs. 51 paired reperfusion (pre- vs. post-LTx) samples from GSE145989 were selected as the training set, and 46 pre- vs. paired post-LTx samples from GSE127003 were selected as the validation set (Wong et al., 2020; Zheng et al., 2022). All of the expression profiles were log2 transformed and normalized using RMA in R (version 3.5.1) with the “affy” package. Boxplots of the normalized expression matrices are shown in Supplementary Figure S2. In addition, CRGs (*n* = 19) were identified using Tsvetkov et al. (2022) and are listed in Supplementary Table S1.

### 2.2 Data processing and identification of DE-CRGs

Datasets were preprocessed using R software (ver. 4.2.2). When multiple probes corresponded to a common gene, the average value was obtained as its expression value using “Limma” (ver. 3.54.2) in the R package. Thereafter, the expression matrix of CRGs was obtained. Finally, DE-CRGs were identified by comparing pre- and post-LTx samples using the Wilcoxon test, and the results were presented with a boxplot and heatmap using “ggpubr” (Ver. 0.6.0) and “pheatmap” (ver. 1.0.12) in the R package. Correlation analysis of DE-CRGs was conducted and visualized through a circle and matrix plot using “corrplot” (ver. 0.92) and “circlize” (ver. 0.4.15) in the R package.

### 2.3 Functional enrichment analysis

Gene Ontology (GO) and Kyoto Encyclopedia of Genes and Genomes (KEGG) analyses were performed using DAVID (https://david.ncifcrf.gov/). The results were downloaded and visualized by “ggplot2” (Ver. 3.4.1) in the R package.

### 2.4 Screening of optimal feature genes (OFGs) and obtaining biomarkers

Three ML methods were used to screen OFGs. LASSO was used to reduce the dimensions of DE-CRGs using “glmnet” (ver. 4.1.4) in the R package, and the optimal penalty parameter λ was obtained by minimal binomial deviance. The point with the smallest cross-validation error was used to screen OFGs with SVM-RFE using “e1071” (Ver. 1.7.11) in the R package. Meanwhile, “randomForest” (ver. 4.7.1.1) in the R package was used to conduct RF to identify the point where the error was minimal, and a MeanDecreaseGini score >2 was regarded as the cutoff value to select OFGs. Finally, biomarker genes were obtained by intersecting these OFGs and visualized using a Venn diagram.

### 2.5 Construction of receiver operating characteristic (ROC) curves

The diagnostic ability of these biomarkers was assessed by constructing receiver operating characteristic (ROC) curves and calculating the area under the curve (AUC) using “pROC” (ver. 1.18.0) in the R package. Furthermore, a logistic regression model (LRM) was constructed to predict the sample types using “glm” (ver. 4.1.4) in the R package, and the diagnostic power of this model was evaluated using ROC curves. GSE127003 was used to verify biomarkers based on the expression differences, ROC curves, and the LRM.

### 2.6 Single-gene gene set enrichment analysis (GSEA)

To explore the function of these biomarkers in post-LTx samples, the R package “clusterProfiler” (Ver. 4.6.2) was used for single-gene GSEA. The post-LTx samples (*n* = 51) were divided into high and low subgroups based on the median expression level of each biomarker, and the “limma” package was applied to calculate the difference in the expression of other genes between different subgroups. Subsequently, all genes were sorted from top to bottom, according to their logFC values, and these sorted genes were regarded as the gene sets to be tested. Meanwhile, “c2.cp.kegg.v2022.1. Hs.symbols.gmt” was used as a predefined set to detect significantly enriched pathways with *p* < 0.05, FDR < 25%, and |NES|>1. The top eight gene sets were visualized using “enrichplot” (ver. 1.18.3) in the R package.

### 2.7 Immune infiltration analysis

Using R software, the proportion of 22 types of immune cells in ALIRI was calculated with CIBERSORT*,* a method used to analyze different immune cell types in tissues (Newman et al., 2015). The proportion of different immune cell types in each sample was calculated and displayed as a bar graph, and the levels of each immune cell type were compared between the post- and pre-LTx groups. The differential immune cells (DICs) were selected with *p* < 0.05 and visualized with a boxplot using the “ggpubr” R package. The results were validated using GSE127003. The Pearson correlation between the 22 infiltrating immune cells and these biomarkers was investigated in the post-LTx samples, and the result was visualized using the “ggplot2” R package.

### 2.8 Construction of the ceRNA network

miRNAs that target the mRNAs of these biomarkers were predicted using miRanda software, TargetScan, and miRDB. Only the miRNAs predicted using the three software programs were included. Subsequently, spongeScan (http://spongescan.rc.ufl.edu) was used to identify long non-coding RNAs (lncRNAs) that bind to the putative miRNA. Finally, the lncRNA–miRNA–mRNA ceRNA regulatory network was constructed and visualized using Cytoscape (version 3.9.1) (https://cytoscape.org).

### 2.9 Development of the rat lung transplantation model

Twelve healthy 11-week-old male Lewis rats (six pairs) were purchased from Beijing Vital River Laboratory Animal Technology Co., Ltd. (Beijing, China). The rats were housed in sterile cages with a humidity of 45%–55% and a light/dark cycle of 12 h and were bred adaptively for 1 week before the initiation of the experiment. This study was reviewed and approved by the Medical Ethics Committee of the Second Affiliated Hospital of Guangxi Medical University [No. 2022-KY(0683)]. Orthotopic left LTx was performed (syngeneic: Lewis to Lewis) using a cuff technique, as previously described (Tian et al., 2020). The donor lungs were flushed and preserved for 6 h with 4°C low potassium dextrose (LPD) before transplantation. The right donor lungs were collected at the end of cold ischemia, and the left donor lungs were obtained after 2 h of reperfusion, following transplantation, and quickly frozen in liquid nitrogen and stored at −80°C.

### 2.10 Quantitative real-time PCR and Western blotting

Total RNA was extracted from frozen lung tissues using TRIzol reagent (Vazyme, China). Script RTase (StarLighter) was used for reverse transcription. The primers used in this study are listed in Table 1. The mRNA levels of these biomarkers were calculated with the 2^-△△Ct^ method and normalized to β-actin. Lung tissues were homogenized with RIPA lysis buffer (Solarbio: #R0010) containing protease inhibitors. The protein concentration was tested using a BCA protein detection kit (Beyotime: #P0010). Equal loading protein was divided by 4%–12% FuturePAGE™ and then transferred to polyvinylidene fluoride (PVDF) membranes. After blocking with 5% skimmed milk, the membranes were hatched with antibodies against NFE2L2 (Proteintech: #16396-1-AP), NLRP3 (Wanleibio: #WL02635), LIPT1 (Aviva Systems Biology: # AAP48784/AAPY02594), and MTF1 (Abmart: MG765438) overnight at 4°C. The membranes were washed with TBST containing Tween and then incubated with corresponding HRP-conjugated secondary antibodies at 37°C for 1 h. Finally, proteins were visualized with a ChemiDoc™ Imaging System. After removing the primary and secondary antibodies with stripping buffer, β-actin (Zenbio:# 38024), which was used as a reference, was detected in the same aforementioned way. The intensity of protein bands was calculated by ImageJ software.

**TABLE 1:** Primer pairs used for qRT-PCR.

Gene symbol	Forward primer (5′->3′)	Reverse primer (5′->3′)
*NLRP3*	TCT​TTG​CGG​CTA​TGT​ACT​ATC​T	TTC​TAA​TAG​GAC​CTT​CAC​GT
*NFE2L2*	TCT​GGA​GAC​GGC​CAT​GAC​TG	AGA​CAC​TGT​AAC​TCG​GGA​ATG​GA
*LIPT1*	TCA​GCA​CAA​GGT​CCC​AAC​AG	CCG​ATG​ACG​ACA​GAA​GGA​GAA​T
*MTF1*	CAT​GAA​AGG​TCA​CGA​TAA​CAA​AGG	GGC​CCA​ACT​CAC​TCA​GAT​AAA
*β-Actin*	TGC​TAT​GTT​GCC​CTA​GAC​TTC​G	GTT​GGC​ATA​GAG​GTC​TTT​ACG​G

### 2.11 Statistical analysis

All statistical analyses and graphics were performed using R software, and statistical significance was set at *p* < 0.05.

## 3 Results

### 3.1 Identification of DE-CRGs

**FIGURE 1:**
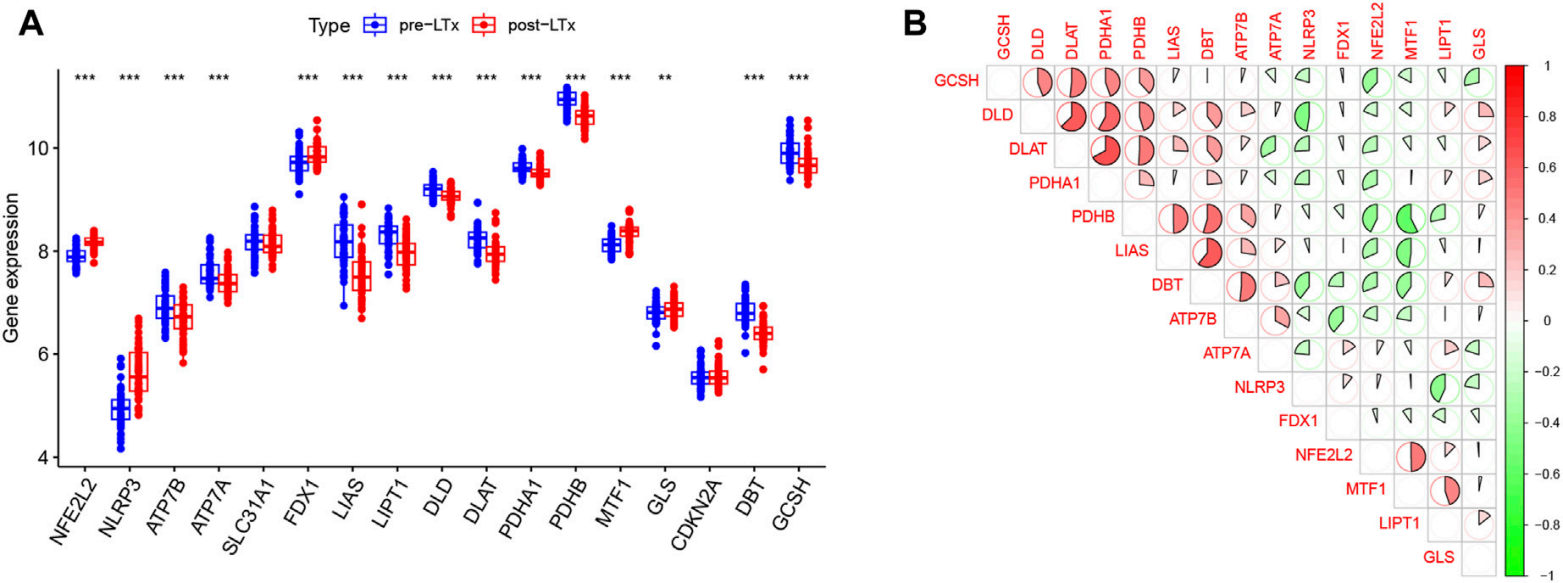
**(A)** In this boxplot, the abscissa represents CRGs and the ordinate represents the expression of genes. * represents *p* < 0.05, ** *p* < 0.01, and *** *p* < 0.001. **(B)** In the matrix plot, the abscissa and diagonals represent the differentially expressed cuproptosis-related gene (DE-CRG) symbols. A positive correlation is shown in red, and a negative correlation in green. Larger sector areas and deeper colors indicate more significant correlations.

Fifteen DE-CRGs were identified, and a boxplot (Figure 1A) and heatmap (Supplementary Figure S3) were generated. After 2 h of reperfusion, we observed the upregulation of genes, including *NFE2L2*, *NLRP3*, *FDX1*, *MTF1*, and *GLS*, and the downregulation of genes, including *ATP7A*, *ATP7B*, *LIAS*, *LIPT1*, *DLD*, *DLAT*, *PDHA1*, *PDHB*, *DBT*, and *GCSH*. Strong positive correlations (r > 0.4) were observed between the following pairs: *NFE2L2* and *MTF1*, *LIAS* and *PDBT*, *LIPT1* and *MTF1*, *DLD* and *PDHB*, *GCSH* and *DLD*, *PDHA1* and *GCSH*, *GCSH* and *DLAT*, *DLD* and *DLAT*, *DLD* and *PDHA1*, *DLAT* and *PDHA1*, *DLAT* and *PDHB*, *PDHB* and *DBT*, *LIAS* and *DBT*, and *DBT* and *ATP7B.* Negative correlations (r < −0.4) were observed between the following pairs: *DLD* and *NLRP3*, *LIPT1* and *NLRP3*, *LIAS* and *MTF1*, and *PDHB* and *MTF1* (Figure 1B).

### 3.2 Functional enrichment analysis of DE-CRGs

**FIGURE 2:**
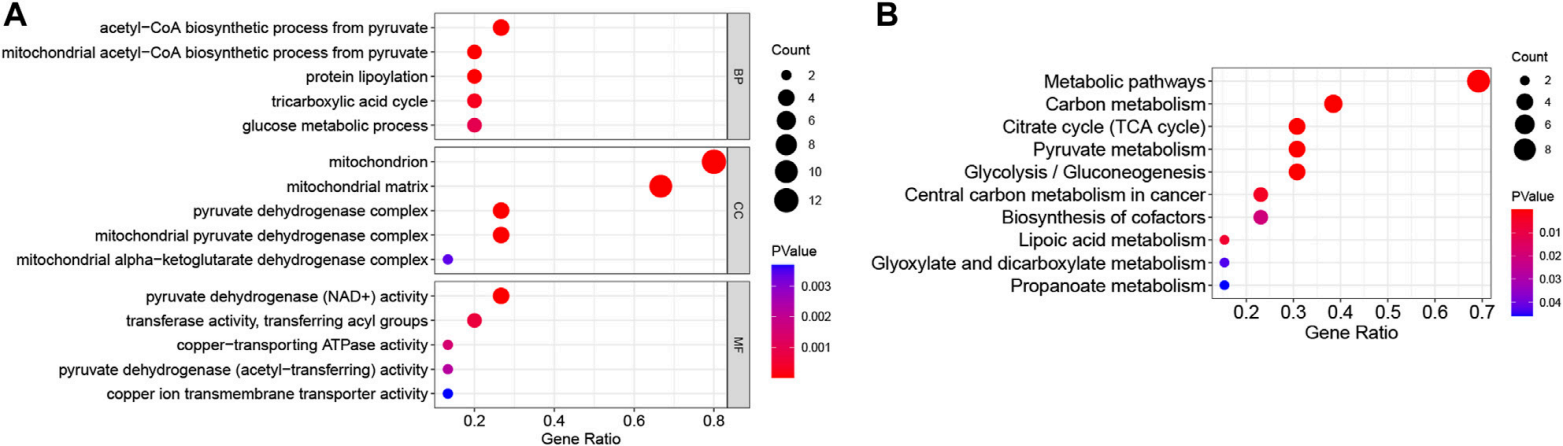
Functional enrichment analysis of DE-CRGs. **(A)** The top five items of Gene Ontology (GO) analysis based on the enriched count of DE-CRGs are shown. **(B)** All Kyoto Encyclopedia of Genes and Genomes (KEGG) pathways are shown. The size of the circles represents the enriched count of DE-CRGs, and a deeper red color indicates a more significant *p*-value. All *p* < 0.05. BP, biological process; CC, cellular component; MF, molecular function.

GO analysis included enriched biological process (BP), cellular component (CC), and molecular function (MF) items, and the top five most significant terms are shown in Figure 2A. In the BP category, DE-CRGs were significantly enriched in the mitochondrial acetyl-CoA biosynthetic process from pyruvate, protein lipoylation, and the TCA cycle; in CC, they were enriched in the mitochondria and pyruvate dehydrogenase complex; and in MF, they were enriched in pyruvate dehydrogenase (NAD+) activity and copper-transporting ATPase activity. In KEGG, DE-CRGs were predominantly enriched in metabolic pathways, carbon metabolism, and the TCA cycle (Figure 2B).

### 3.3 Four DE-CRGs were identified as biomarkers for ALIRI

**FIGURE 3:**
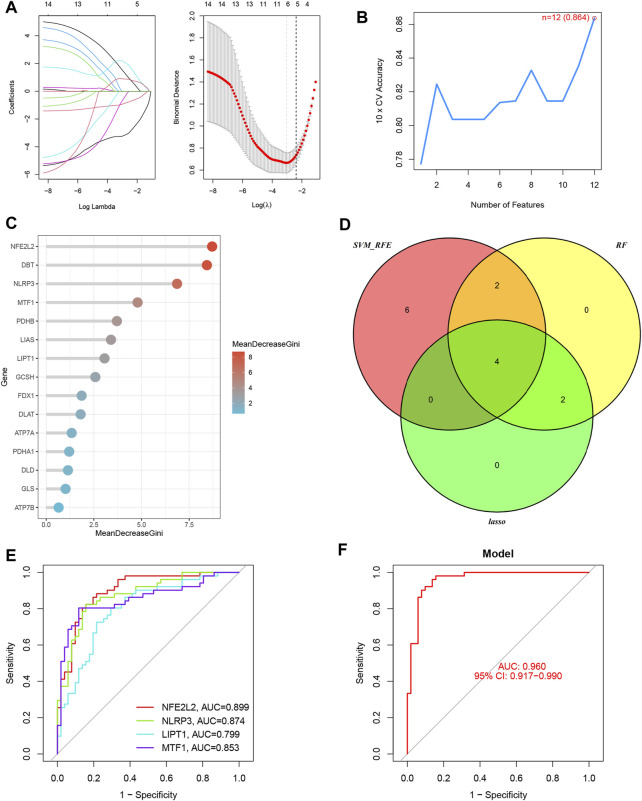
Four biomarkers were identified using three machine learning algorithms. **(A)** Six optimal feature genes (OFGs) were selected from DE-CRGs using least absolute shrinkage and selection operator (LASSO). **(B)** Twelve genes (maximal accuracy = 0.864) were identified as OFGs using support vector machine recursive feature elimination (SVM-REF). **(C)** Eight OFGs (mean decrease Gini score >2) were obtained using random forest (RF). **(D)** The four biomarkers were identified by overlapping these OFGs. **(E)** Receiver operating characteristic (ROC) curves of the individual biomarkers. **(F)** The logistic regression model (LRM) based on the four biomarkers was able to distinguish the post-LTx samples from the pre-LTx samples with an AUC = 0.96.

**FIGURE 4:**
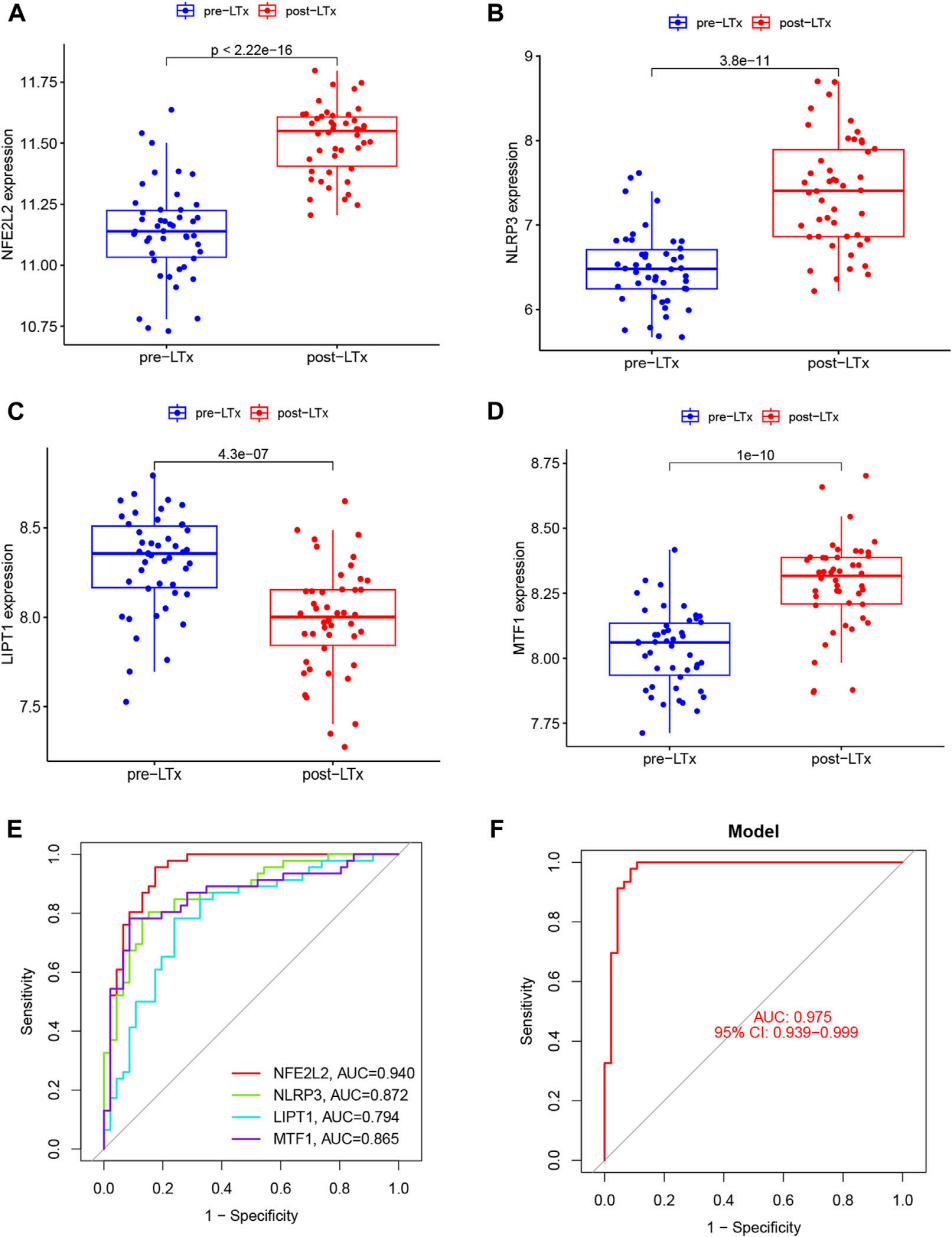
Validation of the biomarkers in GSE127003. **(A–D)** Expression of *NFE2L2*, *NLRP3*, *LIPT1*, and *MTF1* in post-LTx samples compared to pre-LTx samples. **(E)** ROC curves of the predictive efficacy of individual biomarkers. **(F)** LRM based on these four biomarkers distinguished the post-LTx samples from the pre-LTx samples with an AUC = 0.975.

Six OFGs were selected from the DE-CRGs using LASSO (Figure 3A), 12 OFGs (maximal accuracy = 0.864) were selected using SVM-RFE (Figure 3B), and eight OFGs were selected using RF (Figure 3C). These OFGs are listed in Supplementary Table S2. After overlapping OFGs, four biomarkers were identified: *NFE2L2*, *NLRP3*, *LIPT1*, and *MTF1* (Figure 3D). The AUC value was 0.899 for *NFE2L2*, 0.874 for *NLRP3*, 0.799 for *LIPT1*, and 0.853 for *MTF1* (Figure 3E). The ROC curves indicated that the LRM based on the four biomarkers had an excellent ability to distinguish post-LTx samples from pre-LTx samples (AUC = 0.96) (Figure 3F). These biomarkers were verified in the GSE127003 dataset, and their expression levels were similar to those in the training set (Figures 4A–D). In ROC analysis, the AUC values of *NFE2L2*, *NLRP3*, *LIPT1*, and *MTF1* were 0.940, 0.872, 0.794, and 0.865, respectively (Figure 4E). The biomarker-based LRM distinguished post-LTx samples from pre-LTx samples, with an AUC of 0.975 (Figure 4F). These findings demonstrate that these biomarkers have a good diagnostic ability.

### 3.4 Potential function of the biomarkers by single-gene GSEA

The top eight significantly enriched gene set pathways for each biomarker are displayed in Supplementary Figures S4A–D. Genes in the high-expression group of *NFE2L2*, *NLRP3*, and *MTF1* were enriched in 10 common KEGG pathways, which were mainly involved in cell death, immune-related response, and signaling transduction and interaction, including “apoptosis,” “cell adhesion molecules,” “chemokine signaling pathway,” “natural killer cell-mediated cytotoxicity,” “nod-like receptor signaling pathway,” “toll-like receptor signaling pathway,” and “graft-versus-host disease” (Supplementary Table S3). In contrast, genes in the low-expression group of *LIPT1* were enriched in 13 KEGG pathways, which were associated with cell metabolism and proliferation, and signal transduction and interaction (Supplementary Table S4).

### 3.5 Analysis of immune cell infiltration

**FIGURE 5:**
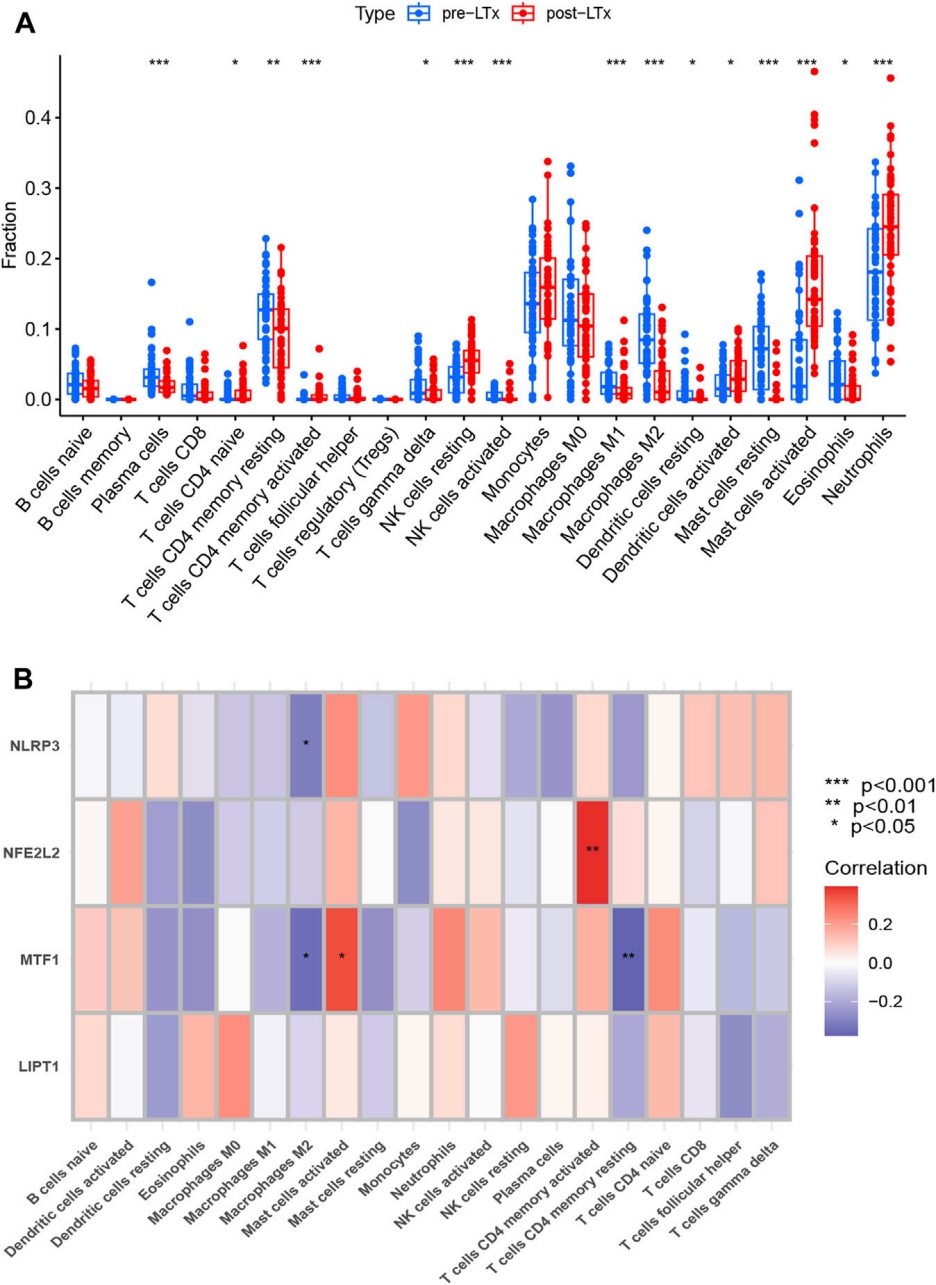
**(A)** Comparison of 22 immune cell subtypes between post-LTx and pre-LTx samples. *p < 0.05, **p < 0.01, and ***p < 0.001. **(B)** Correlation analysis of biomarkers and immune cells. The abscissa displays the name of the immune cells, and the ordinate represents the biomarker genes. Red represents a positive correlation, and blue represents a negative correlation. Deeper colors indicate stronger correlations (*p < 0.05 and **p < 0.01).

The proportions of 22 immune cell types in each sample are shown in Supplementary Figure S5. Compared with the pre-LTx group, neutrophils, activated mast cells (MCs), resting natural killer (NK) cells, and activated dendritic cells (DCs) were upregulated, while plasma cells, resting memory CD4 T cells, M2 macrophages, eosinophils, and resting MCs were downregulated in the post-LTx group (Figure 5A). In the validation set, the proportions of the 22 immune cell types in each sample are shown in Supplementary Figures S6A, and these DICs were found to have the same trends as the training set (Supplementary Figures S6B). Subsequently, we conducted a correlation analysis on the immune cells and the biomarkers and found a positive correlation between *NFE2L2* and activated memory CD4 T cells, and *MTF1* and activated MCs. The following pairs displayed a negative correlation: *MTF1* and resting memory CD4 T cells, *NLRP3* and M2 macrophages, and *MTF1* and M2 macrophages (Figure 5B).

### 3.6 Validation of biomarkers by WB and qRT-PCR in a rat lung transplantation model

**FIGURE 6:**
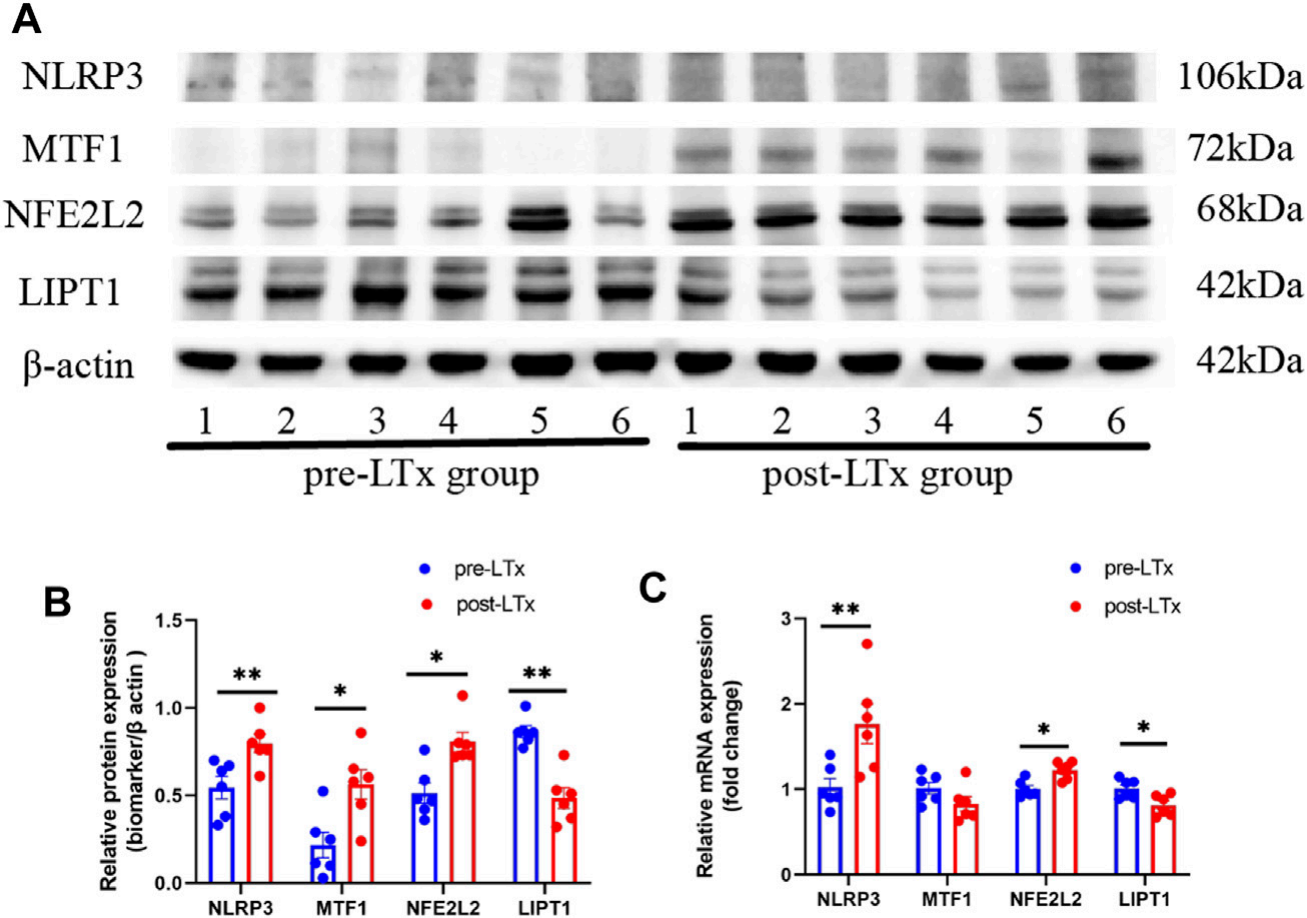
Characterization of these biomarker expression between the post-LTx and pre-LTx groups in rat lung samples. **(A)** Schematic representation of NLRP3, MTF1, NFE2L2, and LIPT1 protein expression by Western blotting, *n* = 6 (pair). **(B)** Statistics of **(A)** plots by ImageJ. **(C)** Relative mRNA expression of *NLRP3*, *MTF1, NFE2L2*, and *LIPT1*. *p < 0.05 and **p < 0.01.

The *NLRP3*, *MTF1*, and *NFE2L2* protein expression levels were significantly higher, and the *LIPT1* protein expression was significantly lower in the post-LTx group than in the pre-LTx group, according to WB (Figures 6A, B). Meanwhile, compared with the pre-LTx group, the relative mRNA expression of *NLRP3* and *NFE2L2* was upregulated, and the relative mRNA expression of *LIPT1* was downregulated in the post-LTx group, which was consistent with what was observed in the dataset analysis. This may be due to the small sample size (*n* = 6) as there was no significant difference in *MTF1* mRNA expression (Figure 6C).

### 3.7 A ceRNA network was established according to the biomarkers

**FIGURE 7:**
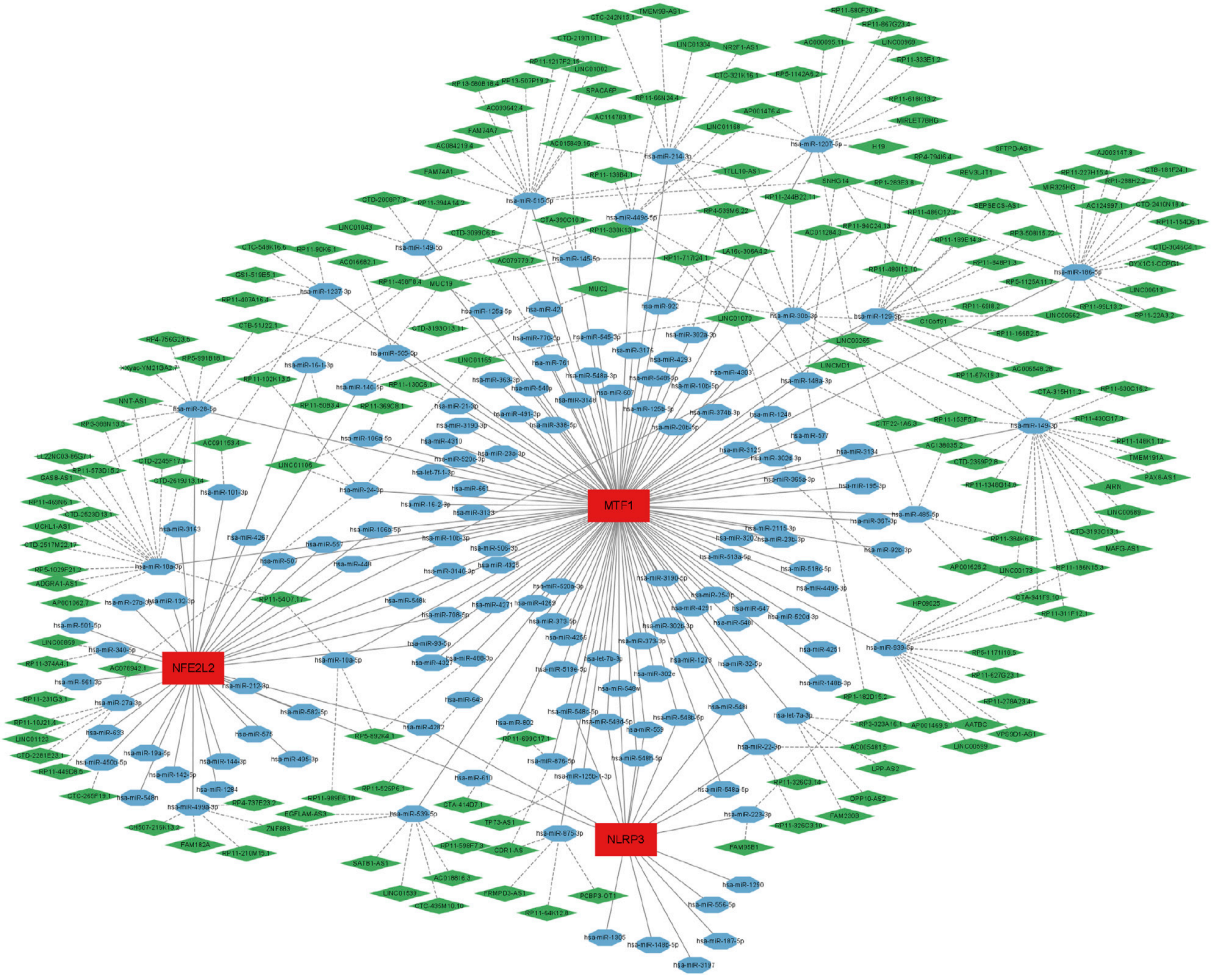
A long non-coding RNA (lncRNA)-miRNA-mRNA competing endogenous RNA (ceRNA) network was established based on *NFE26L2*, *NLRP3*, and *MTF1*. The complex network included 331 nodes (181 lncRNAs, 147 miRNAs, and 3 biomarkers) and 403 edges. The green diamonds represent lncRNAs, blue octagons represent miRNAs, and red rectangles represent mRNAs.

We established a ceRNA network based on three biomarkers (*NFE2L2*, *NLRP3*, and *MTF1*, no *LIPT1*) (Figure 7). The complex network included 331 nodes (181 lncRNAs, 147 miRNAs, and 3 biomarkers) and 403 edges. Specifically, 35, 18, and 115 miRNAs were found to interact with *NFE2L2*, *NLRP3*, and *MTF1*, respectively. Approximately 43 lncRNAs competitively bound to hsa-miR-129-5p, hsa-miR-27a-3p, hsa-miR-28-5p, and hsa-miR-450b-5p and regulated *NFE2L2*; five lncRNAs could regulate the expression of *NLRP3* by competitively binding to hsa-miR-22-3p and hsa-miR-223-3p. For *MTF1*, 214 lncRNAs regulated the expression mainly through competitive binding with hsa-miR-1207-5p, hsa-miR-129-5p, hsa-miR-149-3p, hsa-miR-186-5p, hsa-miR-18a-3p, hsa-miR-214-3p, hsa-miR-28-5p, hsa-miR-30b-3p, hsa-miR-449c-5p, hsa-miR-515-5p, hsa-miR-939-5p, and hsa-miR-539-5p. These results suggest that these biomarkers may play key roles in ALIRI.

## 4 Discussion

In lung transplants, ALIRI is inevitable during organ ischemia, followed by reperfusion. Previous studies have reported cell death to be one of the major mechanisms underlying ALIRI (Capuzzimati et al., 2022). Cuproptosis, a novel type of cell death, is characterized by intracellular free copper overload and lipoylated protein aggregation, leading to cytotoxic stress and cell death (Tsvetkov et al., 2022). However, the role of cuproptosis in ALIRI has not yet been investigated. In the present study, four cuproptosis-related biomarker genes (*NFE2L2*, *NLRP3*, *LIPT1*, and *MTF1*) were identified in the datasets and verified in a rat LTx model. Correlation analysis between these biomarkers and immune infiltration was performed using CIBERSORT*.* ROC curves and LRM analysis revealed that these biomarkers had excellent diagnostic efficiency for ALIRI. Single-gene GSEA indicated that these biomarkers were closely related to pathways involved in cell death, immune-related responses, and signal transduction and interactions.

Fifteen DE-CRGs were identified in the post- and pre-LTx samples. After 2 h of reperfusion, *NFE2L2*, *NLRP3*, FDX1, *MTF1*, and *GLS* were upregulated, whereas *ATP7A*, *ATP7B*, *LIAS*, *LIPT1*, *DLD*, *DLAT*, *PDHA1*, *PDHB*, *DBT*, and *GCSH* were downregulated. FDX1, a mitochondrial matrix enzyme, is a key regulator of copper ionophore-induced cell death, and the deletion of *FDX1* confers cellular resistance to cuproptosis (Tsvetkov et al., 2022). The upregulation of FDX1 enzymatic activity can facilitate elesclomol cytotoxicity by reducing Cu^2+^ to Cu^1+^, resulting in a unique copper-dependent cell death (Tsvetkov et al., 2019). ATP7A and ATP7B are Cu^1+^-transporting ATPases that can translocate Cu^+^ to the extracellular medium when intracellular copper levels are elevated (Tadini-Buoninsegni and Smeazzetto, 2017). In this study, we found that the *FDX1* gene was upregulated and *ATP7A/B* was downregulated after reperfusion, which may result in intracellular free-copper accumulation, leading to cuproptosis. GO analysis indicated that these DE-CRGs were significantly enriched in protein lipoylation, the TCA cycle, pyruvate dehydrogenase (NAD+) activity, and copper-transporting ATPase activity. After KEGG analysis, we found that DE-CRGs were enriched in the TCA cycle. These biological processes are characteristic of cuproptosis and are involved in the pathophysiological mechanisms of ALIRI.

*NFE2L2*, also known as nuclear factor erythroid2-related factor 2 (*Nrf2*), is a master regulator of the redox balance (Bellezza et al., 2018). In IRI, accumulated reactive oxygen species (ROS) result in reduced Nrf2 degradation, leading to Nrf2 upregulation, which strengthens the activity of antioxidant enzymes to reduce ROS production, prevent cell death, and reduce inflammation by decreasing pro-inflammatory factors (Sadrkhanloo et al., 2022). Dong et al. (2020) found that Nrf2 inhibits ferroptosis and protects against acute lung injury (ALI) by regulating *SLC7A11* and heme oxygenase-1 (*HO-1*) (Dong et al., 2020). In addition, Nrf2 can alleviate ALI and inflammation by modulating TLR4 and Akt signaling (Yan et al., 2018). A recent study demonstrated that copper-induced ROS can promote lipid peroxidation, reduce antioxidant enzyme activity, and activate the Nrf2/HO-1 signaling pathway in hippocampal neurons (Lu et al., 2022). A high Cu intake can lead to oxidative damage and activate the Nrf2/HO-1-mediated antioxidant pathway in the porcine myocardium (Li et al., 2022). However, some studies have also shown that administering an optimal level of copper ions has ameliorative effects against IRI in the spinal cord and liver (Tural et al., 2021; Đurašević et al., 2021). An optimal concentration of Cu^2+^ can reduce tissue damage during IR, whereas copper-ion overload can induce cell death (Chen et al., 2023). This difference may be attributed to a difference in tissues. In this study, we found that the expression of *NFE2L2* was upregulated in the post-LTx group using dataset analysis, which was verified by WB and qRT-PCR in a rat LTx model. Recently, Zhao et al. also reported that both the RNA and protein expression of *NFE2L2* are increased in human and mouse ALIRI samples after LTx, which further verifies our findings (Zhao et al., 2023). However, a direct relationship between over-expressed *NFE2L2* and cuproptosis has not been reported. Therefore, we speculate that Cu accumulation induced by IR may further lead to oxidative damage and activate the Nrf2/HO-1-mediated antioxidant pathway. In summary, activated *NFE2L2* can protect against IRI by inhibiting ferroptosis, inflammation, and possibly cuproptosis; however, further investigation is required to confirm this.

*NLRP3* (NOD-like receptor thermal protein domain-associated protein 3), an intracellular signaling molecule, is a member of the NOD-like receptor family, and its inflammasome complex consists of NLRP3, apoptosis-associated speck-like protein containing a CARD (ASC), and pro-cysteinyl aspartate-specific proteinase-1 (pro-caspase-1) (Huang et al., 2021). *NLRP3* is activated by a variety of pathogen- and host-derived chemical factors through complex mechanisms (Xue et al., 2019). *NLRP3* is upregulated and activated in murine lung IR, and the selective inhibition of the NLRP3 inflammasome attenuates IRI (Xu et al., 2018). Additionally, rat LTx research has shown that the NLRP3 protein is increased in the grafted lung after 2 h of reperfusion, and ozone can protect against ALIRI by attenuating *NLRP3*-mediated inflammation and enhancing Nrf2 antioxidant activity (Zheng et al., 2021). Furthermore, *NLRP3* knockout not only reduces cerebral IR injury but also alleviates the severity of cerebral IR-induced lung injury (Xu et al., 2022). Some substances, such as dexmedetomidine and corilagin, can alleviate IR-induced lung injury by inhibiting NLRP3 inflammasome activation (Chen et al., 2020; Li et al., 2022). Recent studies have shown that activation of the NLRP3 inflammasome contributes to several types of cell death, including pyroptosis, apoptosis, necroptosis, and ferroptosis (Huang et al., 2021). However, there have been no reports on the relationship between *NLRP3* and cuproptosis in patients with IRI. Deigendesch et al. found that Cu is required for NLRP3 inflammasome activation and that depletion of bioavailable Cu can reduce inflammasome activation (Deigendesch et al., 2018). In our study, the protein and mRNA levels of *NLRP3* were overexpressed in both the human dataset and the rat LTx experiment, and this gene was selected as a biomarker for ALIRI. Controlling Cu ions and targeting *NLRP3* may be an optional therapy for ALIRI.

Acyltransferase 1, encoded by *LIPT1*, transfers lipoic acid to the E2 subunit of α-ketoglutarate dehydrogenase (Mayr et al., 2014). Thus, a deficiency of the *LIPT1* homolog can lead to reduced lipoylation of E2 subunits (Lv et al., 2022). Lipoic acid is an essential co-factor for α-ketoglutarate dehydrogenase and the glycine cleavage system in mitochondria, which is closely related to the TCA cycle, and *LIPT1* deficiency can suppress the TCA cycle (Fan et al., 2022; Solmonson et al., 2022). LIPT1 supports lipogenesis and balances oxidative and reductive glutamine metabolism (Ni et al., 2019). Our study revealed that the protein and mRNA expression levels of *LIPT1* were lower in the post-LTx group than in the pre-LTx group via dataset analysis and animal experiments, which is consistent with the findings in a cerebral infarction model (Fan et al., 2022). Single-gene GSEA indicated that 13 enrichment pathways were activated in the low-expression *LIPT1* group and were involved in the cell cycle, cell metabolism, and signal transduction and interaction. Therefore, we hypothesized that in ALIRI, downregulated *LIPT1* could restrain the TCA cycle and result in imbalanced oxidative and reductive glutamine metabolism, leading to cell death. Cuproptosis occurs through the direct binding of copper to the lipoylated components of the TCA cycle (Tsvetkov et al., 2022). However, the regulatory mechanism of *LIPT1* in cuproptosis requires further investigation.

MTF1 (*m*etal-regulatory transcription factor 1) is a conserved metal-binding transcription factor that can bind metal-responsive elements (MREs) to drive the transcription of target genes that maintain metal homeostasis, including Zn and copper homeostasis (Chen et al., 2020). When the intracellular copper ion concentration is elevated, MTF1 activates metallothioneins to protect the cell, while the transporter ATP7 removes excess copper from the cell by changing its subcellular localization. Under low copper conditions, MTF-1 activates the copper importer Ctr1B, and copper is taken up (Balamurugan and Schaffner, 2006). MTF1 can also regulate ATP7B expression by binding to the MRE of the ATP7B promoter (Stalke et al., 2020), which may help regulate the intracellular Cu balance. A recent study showed that Cu recruits MTF-1 to the MRE of the Nrf2 promoter and promotes Nrf2 over-expression (Zhong et al., 2023). In our study, the expression of *NFE2L2* (*Nrf2*) and *MTF1* was upregulated post-LTx, and *NFE2L2* had a strong positive correlation with *MTF1* (r = 0.5). Therefore, we speculate that Cu could also promote *NFE2L2* upregulation via *MTF1* binding to the MRE site of the Nrf2 promoter in ALIRI. In summary, *MTF1* may be a protective factor that maintains the intracellular copper ion balance, induces anti-oxidative damage, and prevents cell death by upregulating *NFE2L2*.

Based on differences in immune infiltration, we found that in post-LTx patients, neutrophils, activated MCs, resting NK cells, and activated DCs increased, but the infiltration of plasma cells, resting memory CD4 T cells, M2 macrophages, eosinophils, and resting MCs decreased. Neutrophil infiltration is a hallmark of ALIRI and promotes allograft injury (Scozzi et al., 2017). In mouse LTx, neutrophils can be rapidly recruited to the allograft depending on the number of monocytes after reperfusion (Kreisel et al., 2010), and TLR4 expression in vascular endothelial cells regulates neutrophil recruitment after IRI (Li et al., 2022). Activated MCs can release a number of mediators, including interleukin (IL)-2, IL-7, IL-3, IL-6, IL-9, IL-10, tumor necrosis factor (TNF)-α, and chemokines, which are involved in graft injury and rejection (Mortaz et al., 2018). In mouse lungs, MCs can promote IRI by increasing the production of prostaglandin D2, TNF-a, and IL-6, which, in turn, is responsible for recruiting neutrophils (Wingard et al., 2011). Using MC stabilizers, lung IRI can be improved by inhibiting the expression of MC-derived ICAM-1 (Vural et al., 2000). Therefore, activated MCs may serve as a therapeutic target in ALIRI. A previous study indicated that NK cells were increased in the lung tissue of an IRI mouse model and seven clinical LTx patients with PGD, and IRI was attenuated in NK cell-deficient mice but restored after the adoptive transfer of NK cells (Calabrese et al., 2021). After LTx, most macrophages in the graft are donor-derived (Kopecky et al., 2020). Macrophages play a fundamental role in ALIRI and are mainly activated during cold preservation and the early (30 min) phases of reperfusion injury, and contribute to local and subsequent inflammation via the induction of cytokines (Fiser et al., 2001; Kopecky et al., 2020). Under different stimuli, macrophages can be polarized into M1 (classical) and M2 (alternative) phenotypes; the former are responsible for pro-inflammatory reactions, whereas the latter play an important role in anti-inflammatory reactions (Sica and Mantovani, 2012). In ALI, the M1 phenotype is involved in the acute phase, whereas M2 is mainly related to the recovery phase of inflammation (Johnston et al., 2012), which is consistent with our finding suggesting that the M2 phenotype was significantly lower after 2 h of reperfusion. We also found that M2 macrophages were negatively correlated with *NLRP3* (r = −0.31). M2 macrophage-derived exosomes can mitigate myocardial IRI by inactivating the TLR4/NF-κB/NLRP3 inflammasome signaling pathway (Dai et al., 2020). Therefore, we speculate that the upregulation of M2 macrophages may improve ALIRI by inhibiting the *NLRP3* inflammasome, and this may provide new directions for research on the treatment of ALIRI. However, some studies also discovered that M2 macrophages may promote the differentiation of lung fibroblasts and cause pulmonary fibrosis; however, the underlying mechanism was found to be complicated and unclear (Cheng et al., 2021). Thus, the role of macrophages in ALIRI needs to be further explored. A rat LTx study discovered that recipient T cells were activated and infiltrated the graft lung rapidly during the early phase of reperfusion (de Perrot et al., 2003). In contrast, in our analysis, we found that the proportion of resting memory CD4 T cells was reduced, and no obvious changes were observed in other types of T cells, which may be attributed to the extensive use of immunosuppressive agents during the perioperative period. In addition, the correlation analysis of immune cells and biomarkers revealed that *MTF1* had a positive correlation with activated MCs (r = 0.35) and a negative correlation with resting memory CD4 T cells (r = −0.37) and M2 (−0.34); however, the underlying mechanism needs to be further explored. We also found that *NFE2L2* had a positive correlation with activated memory CD4 T cells (r = 0.40). Zagorski et al. demonstrated that *NFE2L2* activation inhibits the secretion of the Th1 cytokine IFNγ and promotes Th2 differentiation, thereby initiating an anti-inflammatory response (Zagorski et al., 2018). Furthermore, Noel et al. discovered that T-cell-specific activation of *NFE2L2* can protect from IR-induced AKI in a mouse model (Noel et al., 2015). Therefore, we hypothesize that the upregulation of *NFE2L2* may direct memory CD4 T cells toward Th2 differentiation and induce an anti-inflammatory response in ALIRI. This may be another mechanism through which *NFE2L2* protects against ALIRI. Although most of the aforementioned studies were conducted in animals, our findings were confirmed in clinical LTx patients.

Finally, we analyzed the ceRNA network of biomarker genes. miRNAs play important roles in the development of IRI (Kura et al., 2020). In lung IRI, miR-18a-5p regulates cellular ROS levels and affects the nuclear localization of NFE2L2 into the nucleus (Xiao et al., 2021). Ding et al. demonstrated that the inhibition of miR-29a can ameliorate myocardial IRI by reducing oxidative stress and NLRP3-mediated pyroptosis (Ding et al., 2020). MiR-148a, derived from exosomes of M2 macrophages, can also improve myocardial IRI by inactivating the TLR4/NF-κB/NLRP3 inflammasome signaling pathway (Dai et al., 2020). In our study, five lncRNAs regulated the expression *MTF1* by competitively binding with hsa-miR-148a-3p. However, whether our predicted non-coding RNA plays a role in ALIRI is unclear and requires further experimental verification.

To the best of our knowledge, this is the first study to investigate the role of cuproptosis in ALIRI. Similar to other studies, this study has some limitations. First, the number of transcriptome datasets used was limited. In this study, we analyzed only one training dataset and one validation set. Second, the datasets were derived from human samples but were validated with rat samples, and it might be more beneficial to confirm this using clinical samples in the future.

## 5 Conclusion

Fifteen DE-CRGs and four biomarker genes (*NFE2L2*, *NLRP3*, *LIPT1*, and *MTF1*) identified via bioinformatic analysis and three ML algorithms were closely associated with the pathogenesis of cuproptosis in ALIRI. ROC curves and LRM analysis revealed that these biomarkers exhibited excellent diagnostic efficiency for ALIRI and may serve as predictors of cuproptosis. Subsequent analysis of these genes helped uncover the potential biological pathways and mechanisms of cuproptosis in ALIRI. We performed a differential analysis of immune cell infiltration between post- and pre-LTx samples, followed by correlation analysis between biomarkers and 22 immune cells to obtain DICs. These results provide a certain reference value for basic research on cuproptosis and immune cell infiltration in ALIRI.

## Data Availability

Publicly available datasets were analyzed in this study. These data can be found at: https://www.ncbi.nlm.nih.gov/geo/query/acc.cgi?acc=GSE127003, https://www.ncbi.nlm.nih.gov/geo/query/acc.cgi?acc=GSE145989.
