# Supplementary material for: Identification of cuproptosis-related biomarkers and analysis of immune infiltration in allograft lung ischemia-reperfusion injury
Source: Front Mol Biosci. 2023 Nov 21;10:1269478. doi: 10.3389/fmolb.2023.1269478 (PMC10703368; doi:10.3389/fmolb.2023.1269478)
Supplement: Supplementary file 1 [file DataSheet1.docx]

Supplementary Material

# Supplementary Figures


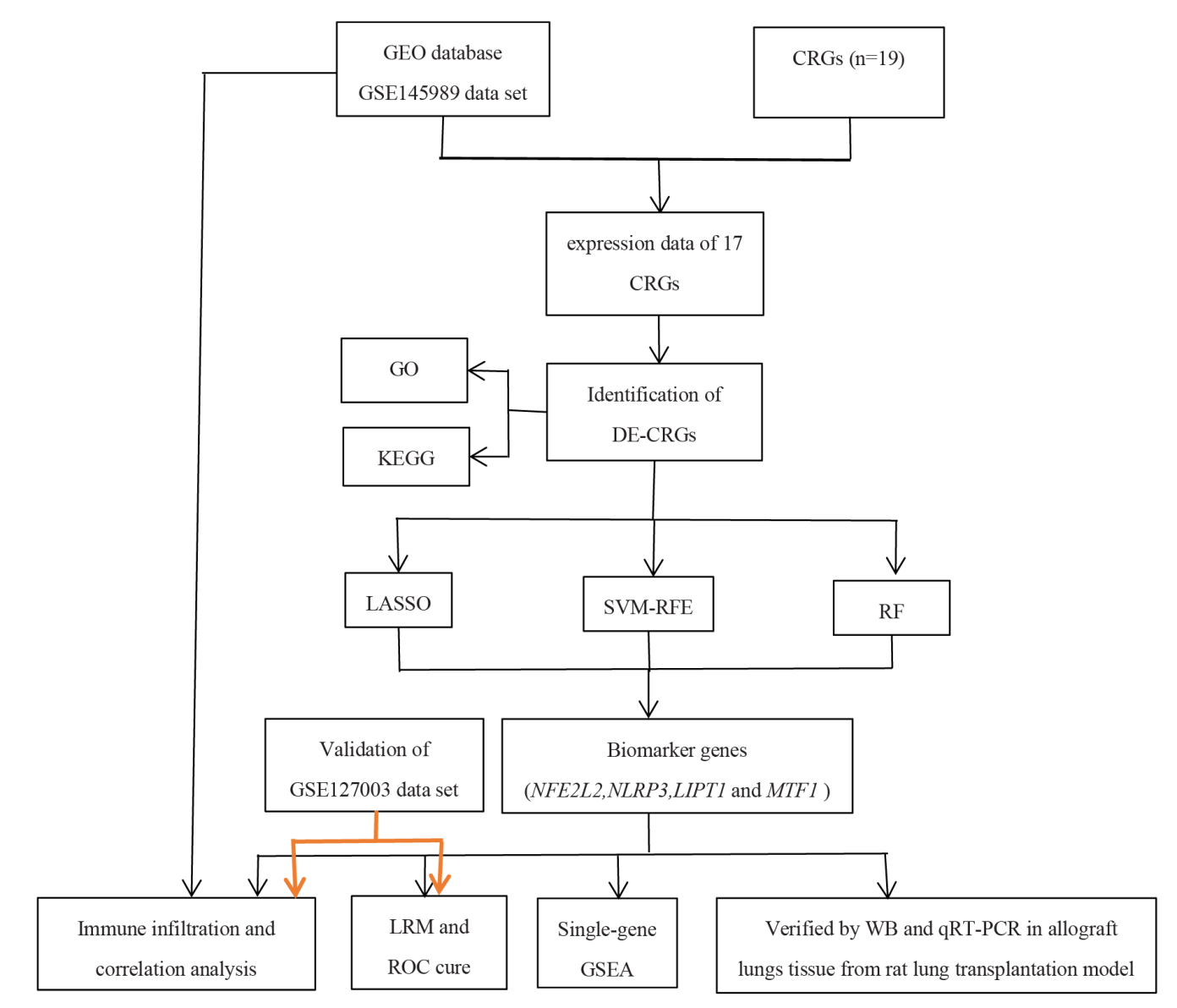


**Supplementary Figure S1. Workflow of the study**. CRGs, cuproptosis-related genes; DE-CRGs, differentially expressed cuproptosis-related genes; GO, Gene Ontology; KEGG, Kyoto Encyclopedia of Genes and Genomes; LASSO, least absolute shrinkage and selection operator; SVM-RFE, support vector machine recursive feature elimination; RF, random forest; LRM, logistics regression model; ROC, receiver operating characteristic; GSEA, Gene Set Enrichment Analysis; WB, Western blotting; qRT-PCR, quantitative real-time PCR .


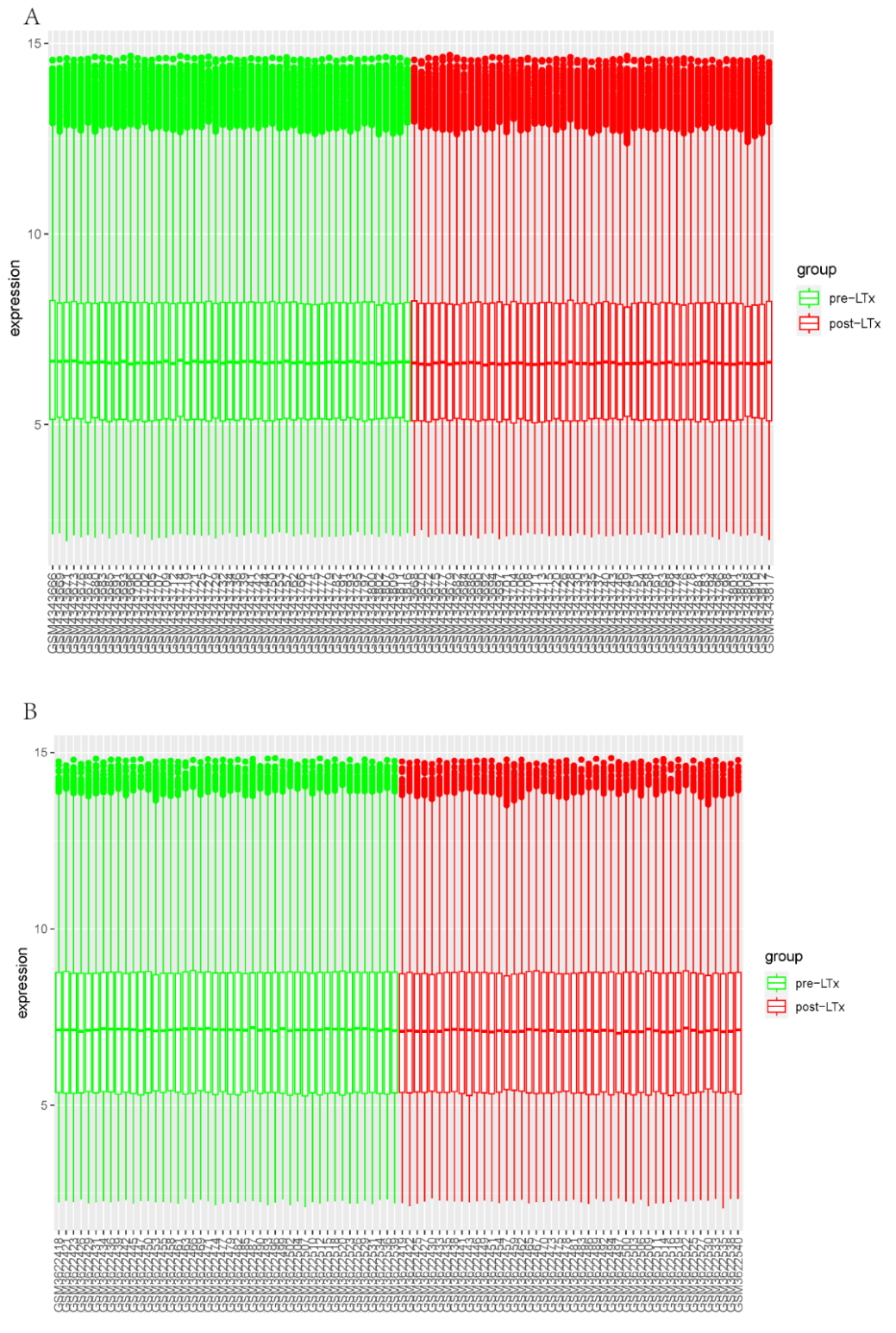


**Supplementary Figure S2.** The boxplots of gene expression data in two datasets after normalization. (A).GSE145989 dataset. (B).GSE127003 dataset


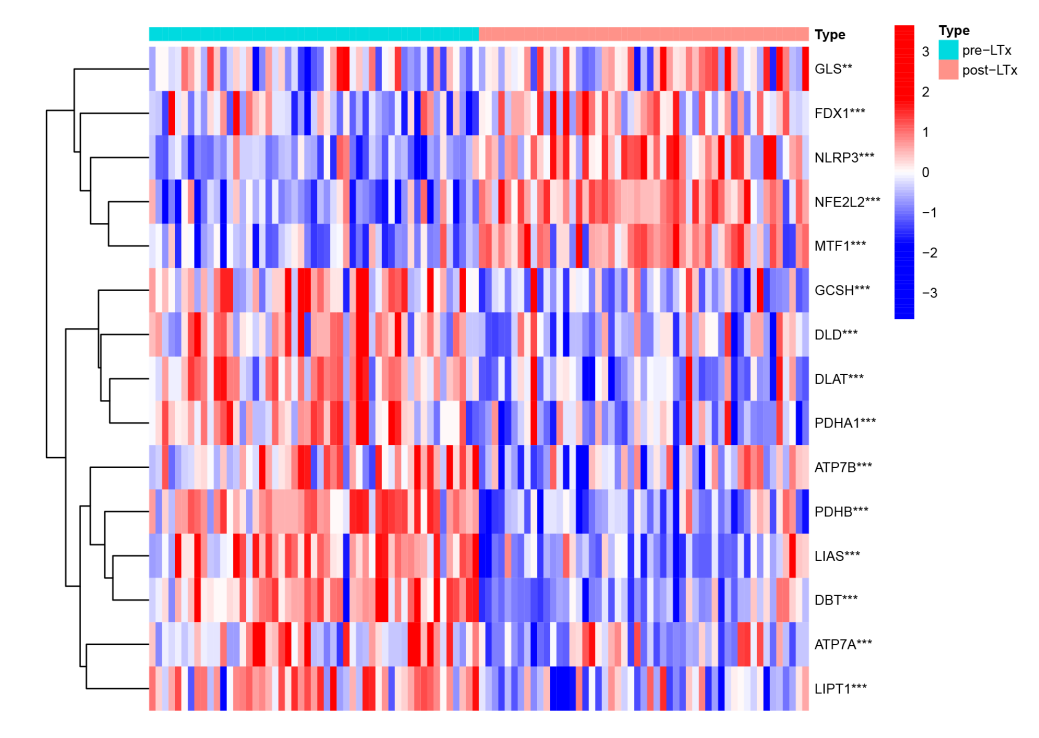


**Supplementary Figure S3.** The heatmap for differential analysis of CRGs. In the heatmap, the abscissa is the sample, and the ordinate is the CRGs. Red represents genes with high expression, and blue represents genes with low expression. * represents P < 0.05, ** P < 0.01 and *** P < 0.001.


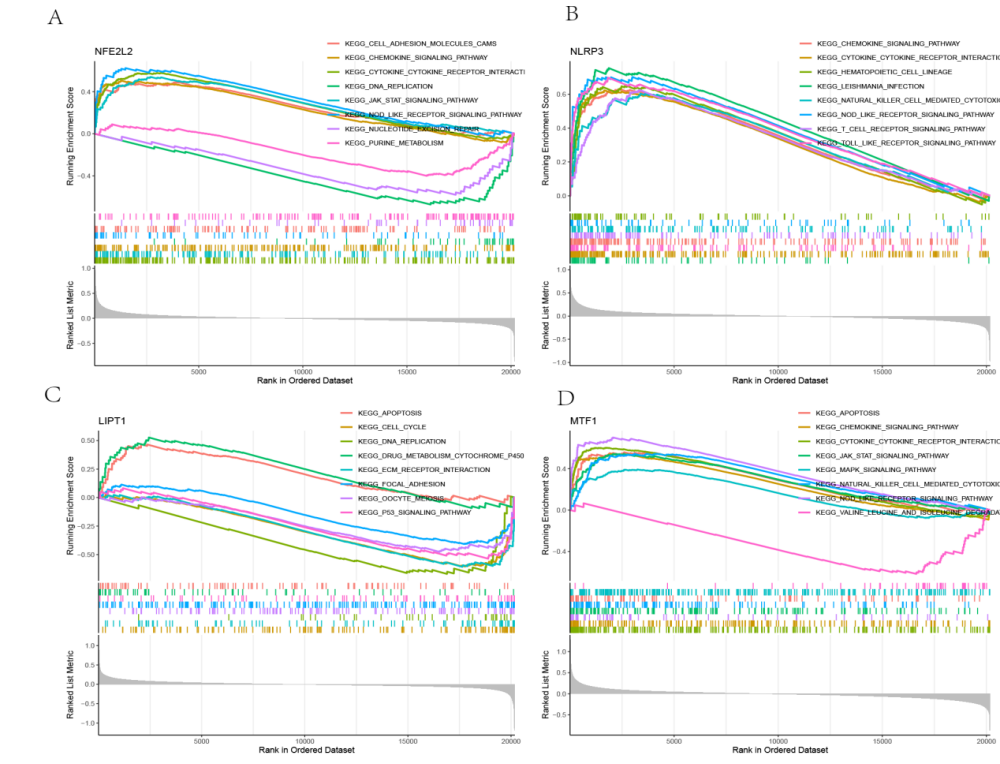


**Supplementary Figure S4.** Single-gene gene-set enrichment analysis (GSEA)-KEGG pathway analysis on *NFE2L2* (A), *NLRP3* (B), *LIPT1* (C), and *MTF1*(D) in the post-LTx samples.


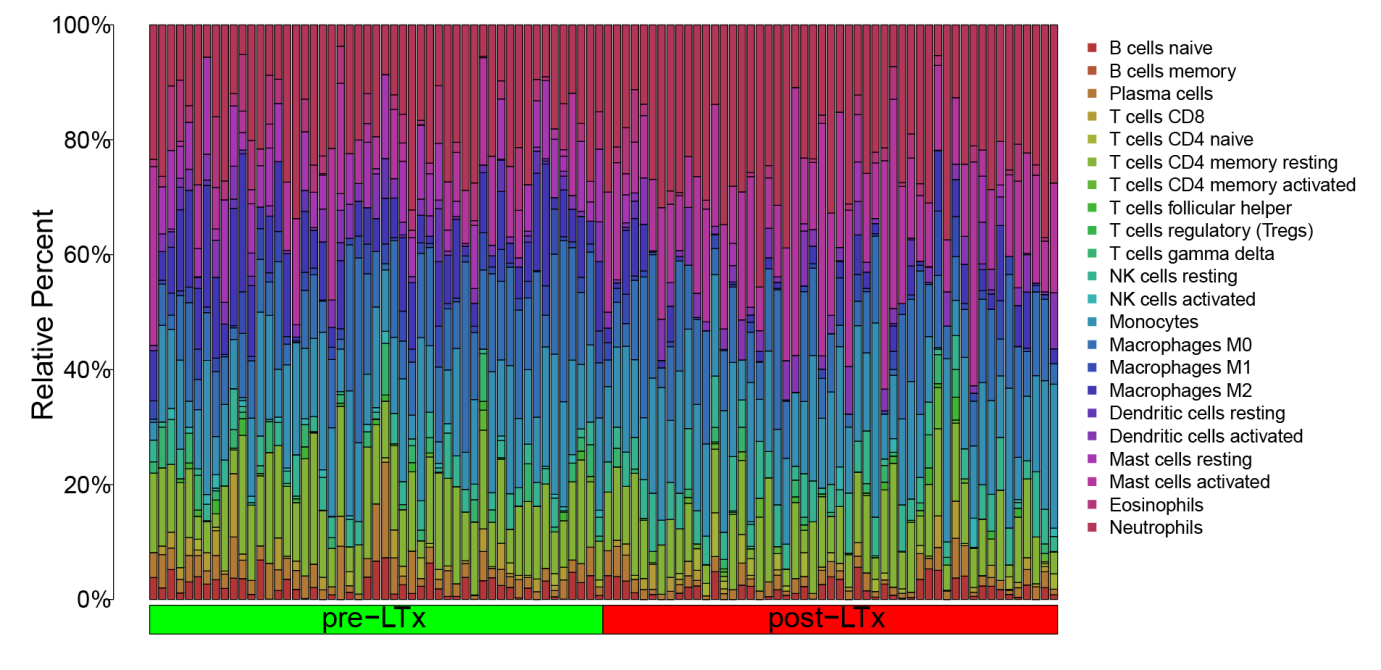


**Supplementary Figure S5.** Analysis of immune infiltration in the post-LTx and pre-LTx group from training dataset. Relative percentages of 22 immune cells in the post-LTx and pre-LTx group.


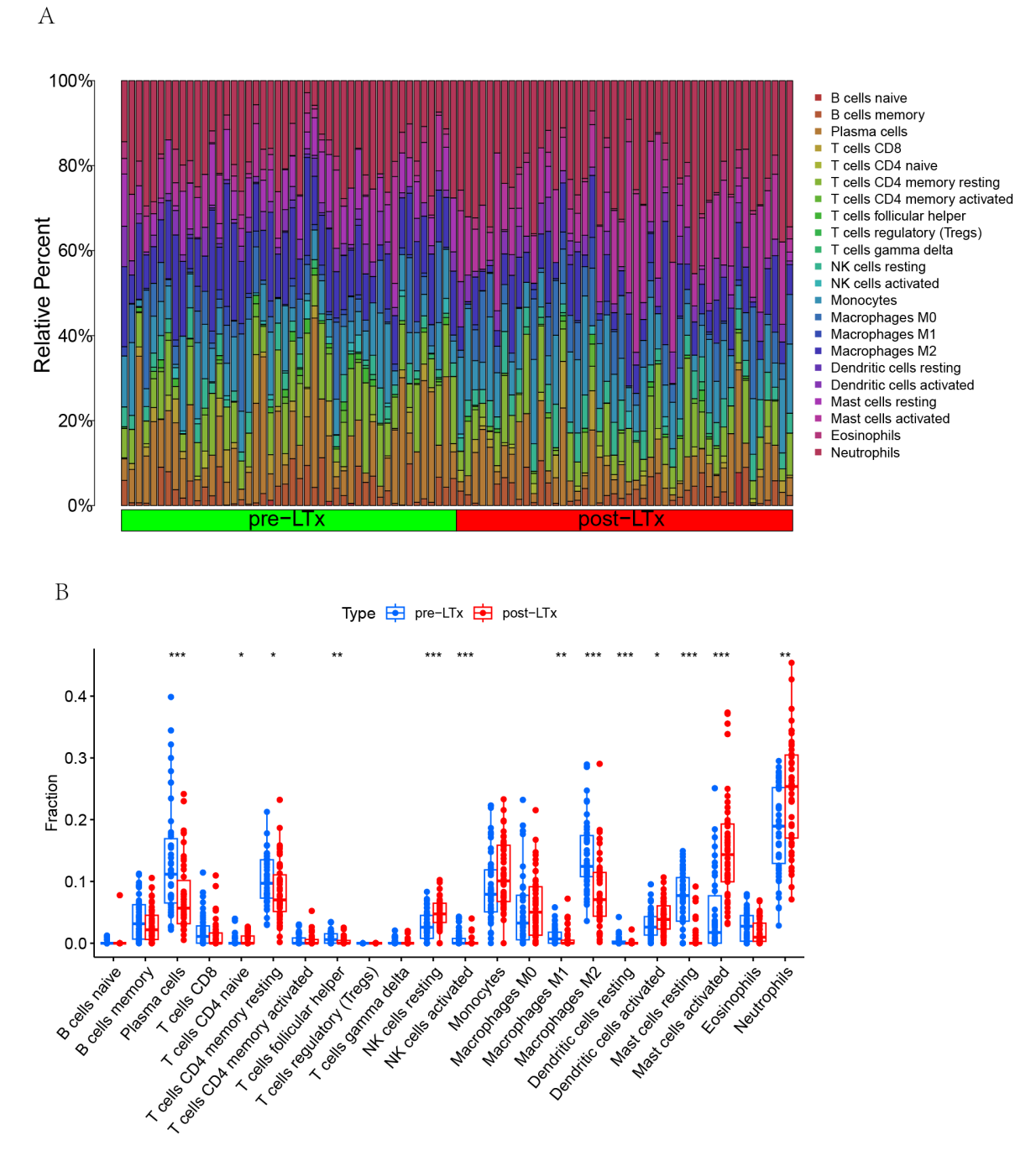


**Supplementary Figure S6.** Analysis of immune infiltration in the post-LTx and pre-LTx group from GSE127003 dataset. (A) Relative percentages of 22 immune cells in the post-LTx and pre-LTx group. (B) Comparison of 22 immune cell subtypes between post-LTx and pre-LTx samples. *P < 0.05, **P < 0.01 and ***P < 0.001.

# Supplementary Tables

**Supplementary Table S1**. Cuproptosis-related Genes

| Gene Symbol | NFE2L2, NLRP3, ATP7B, ATP7A, SLC31A1, FDX1, LIAS, LIPT1, LIPT2, DLD, DLAT, PDHA1, PDHB, MTF1, GLS, CDKN2A, DBT, GCSH, DLST |
| --- | --- |

**Supplementary Table S2.** The optimal feature genes (OFGs) were obtained from least absolute shrinkage and selection operator (LASSO), support vector machine recursive feature elimination (SVM-RFE) and random forest (RF) algorithms.

| Algorithms | Gene symbol (OFGs) |
| --- | --- |
| LASSO (6) | *NFE2L2, NLRP3,* *LIPT1, PDHB, MTF1, DBT* |
| SVM-RFE (12) | *LIPT1, NFE2L2,* *MTF1, NLRP3, FDX1, ATP7A, PDHA1, DLD, LIAS, DLAT, GLS, GCSH* |
| RF (8) | *NFE2L2, DBT, NLRP3, MTF1, PDHB, LIAS, LIPT1, GCSH* |

**Supplementary Table S3.** In the post-LTx samples of GSE145989 data set, genes in high-expression group of *NFE2L2*, *NLRP3* and *MTF1* were enriched in 10 common KEGG pathways (P<0.05, NES>1).

| KEGG Pathway | NES (*MTF1*) | Pvalue (*MTF1*) | NES (*NLRP3*) | Pvalue (*NLRP3*) | NES (*NFE2L2*) | Pvalue (*NFE2L2*) |
| --- | --- | --- | --- | --- | --- | --- |
| APOPTOSIS | 2.09 | 1.82E-06 | 1.59 | 6.35E-03 | 1.83 | 3.88E-04 |
| CELL_ADHESION_MOLECULES_CAMS | 1.55 | 4.47E-03 | 1.78 | 1.42E-04 | 1.89 | 5.23E-05 |
| CHEMOKINE_SIGNALING_PATHWAY | 2.27 | 1.00E-10 | 2.50 | 1.00E-10 | 2.03 | 2.54E-07 |
| CYTOKINE_CYTOKINE_RECEPTOR_INTERACTION | 2.63 | 1.00E-10 | 2.56 | 1.00E-10 | 2.42 | 1.00E-10 |
| CYTOSOLIC_DNA_SENSING_PATHWAY | 1.85 | 1.27E-03 | 2.05 | 3.35E-05 | 1.73 | 4.69E-03 |
| GRAFT_VERSUS_HOST_DISEASE | 1.88 | 1.47E-03 | 2.23 | 4.77E-07 | 1.94 | 6.07E-04 |
| JAK_STAT_SIGNALING_PATHWAY | 2.23 | 1.00E-09 | 1.70 | 4.82E-04 | 2.13 | 4.80E-08 |
| NATURAL_KILLER_CELL_MEDIATED_CYTOTOXICITY | 2.18 | 2.70E-08 | 2.29 | 3.91E-10 | 1.55 | 6.08E-03 |
| NOD_LIKE_RECEPTOR_SIGNALING_PATHWAY | 2.46 | 7.39E-10 | 2.38 | 2.57E-09 | 2.14 | 2.85E-06 |
| PATHOGENIC_ESCHERICHIA_COLI_INFECTION | 2.01 | 5.90E-05 | 1.79 | 1.41E-03 | 1.62 | 7.70E-03 |

**Supplementary Table S4.** In the post-LTx samples of GSE145989 data set, genes in low-expression group of *LIPT1* are enriched in 13 KEGG pathways (P<0.05, NES<-1).

| KEGG Pathway | NES(LIPT1) | pvalue(LIPT1) |
| --- | --- | --- |
| CELL_CYCLE | -2.29 | 9.27E-10 |
| ECM_RECEPTOR_INTERACTION | -2.17 | 2.58E-07 |
| DNA_REPLICATION | -2.04 | 5.24E-05 |
| OOCYTE_MEIOSIS | -1.78 | 1.63E-04 |
| FOCAL_ADHESION | -1.64 | 1.33E-04 |
| P53_SIGNALING_PATHWAY | -1.85 | 5.06E-04 |
| LYSOSOME | -1.69 | 8.46E-04 |
| STEROID_BIOSYNTHESIS | -1.89 | 1.29E-03 |
| TOLL_LIKE_RECEPTOR_SIGNALING_PATHWAY | -1.65 | 1.31E-03 |
| N_GLYCAN_BIOSYNTHESIS | -1.77 | 2.20E-03 |
| LEISHMANIA_INFECTION | -1.70 | 2.87E-03 |
| PURINE_METABOLISM | -1.56 | 3.26E-03 |
| GLYCOSAMINOGLYCAN_BIOSYNTHESIS_CHONDROITIN_SULFATE | -1.72 | 3.80E-03 |
